# Retrospective Analysis of Vaccinated and Unvaccinated COVID-19 Patients Treated with Monoclonal Antibodies (mAb) and Their Emergent Needs (RAVEN)

**DOI:** 10.3390/vaccines11030688

**Published:** 2023-03-17

**Authors:** Gordana Simeunovic, James Polega, Subhan Toor, Nicholas J. Andersen

**Affiliations:** 1 Community Response Department, Spectrum Health, Grand Rapids, MI 49503, USA; 2 Department of Infectious Disease, Spectrum Health, Grand Rapids, MI 49503, USA; 3 College of Human Medicine, Michigan State University, Grand Rapids, MI 49503, USA; 4 Spectrum Health, Infectious Disease Fellowship, Michigan State University, Grand Rapids, MI 49503, USA; 5 Spectrum Health, Internal Medicine Residency, Michigan State University, Grand Rapids, MI 49503, USA; 6 Office of Research and Education, Spectrum Health, Grand Rapids, MI 49503, USA

**Keywords:** COVID-19, monoclonal antibody, vaccine

## Abstract

Strategies to combat COVID-19 include vaccines and Monoclonal Antibody Therapy. While vaccines aim to prevent development of symptoms, Monoclonal Antibody Therapy aims to prevent the progression of mild to severe disease. An increasing number of COVID-19 infections in vaccinated patients raised the question of whether vaccinated and unvaccinated COVID-19 positive patients respond differently to Monoclonal Antibody Therapy. The answer can help prioritize patients if resources are scarce. We performed a retrospective study to evaluate and compare the outcomes and risks for disease progression between vaccinated and unvaccinated COVID-19 patients treated with Monoclonal Antibody Therapy by measuring the number of Emergency Department visits and hospitalizations within 14 days as well as the progression to severe disease, defined through the Intensive Care Unit admissions within 14 days, and death within 28 days from the Monoclonal Antibody infusion. From 3898 included patients, 2009 (51.5%) were unvaccinated at the time of Monoclonal Antibody infusion. Unvaccinated patients had more Emergency Department visits (217 vs. 79, *p* < 0.0001), hospitalizations (116 vs. 38, *p* < 0.0001), and progression to severe disease (25 vs. 19, *p* = 0.016) following treatment with Monoclonal Antibody Therapy. After adjustment for demographics and comorbidities, unvaccinated patients were 2.45 times more likely to seek help in the Emergency Department and 2.70 times more likely to be hospitalized. Our data suggest the added benefit between the COVID-19 vaccine and Monoclonal Antibody Therapy.

## 1. Introduction

COVID-19, a novel viral respiratory disease, quickly reached pandemic status following its identification [1]. As a result, the U.S. government declared a public health emergency on 31 January 2020 [2]. In response, the Department of Health and Human Services released an Emergency Use Authorization (EUA) declaration to warrant the use of unapproved diagnostics and therapeutics in controlled settings [3]. Over the next six months, antiviral medications received EUAs to treat severely ill hospitalized COVID-19 patients [4]. However, at that time, outpatient treatment options remained limited.

The acceleration of cases in the fall of 2020 placed further pressure on medical authorities to identify efficacious treatments to reduce immense COVID-19 related morbidity and mortality. In November 2020, the United States Food and Drug Agency (FDA) granted EUAs for Monoclonal Antibodies (mAb), the first outpatient COVID-19 therapy [5]. mAb imitate natural monoclonal IgG antibodies and bind non-competitively to the SARS-CoV-2 spike protein receptor, blocking the ability of the virus to enter human cells [6]. Bamlanivimab was the first to receive EUA in November 2020 [5], followed by casirivimab–imdevimab (November 2020) [6], bamlanivimab–etesevimab (February 2021) [7], and sotrovimab (May 2021) [8]. Under the EUA guidelines, mAb were authorized to treat non-hospitalized patients with mild to moderate disease with risk factors for disease progression within ten days of the symptom onset [5,6,7,8]. Patients with mild-to-moderate COVID-19 who received any of the authorized mAb had a lower frequency of Emergency Department (ED) visits, COVID-19 related hospitalizations, and all-cause mortality compared to placebo [9,10,11]. In December 2020, FDA granted EUAs to three different COVID-19 vaccines [12,13,14]. Vaccines reduce the risk of acquiring COVID-19, particularly severe disease, as well as COVID-19 related morbidity and mortality [15,16,17].

While vaccination and mAb are independently associated with reduced hospitalization and all-cause mortality in COVID-19 positive patients, and mAb therapy further decreases the risk of disease progression in vaccinated patients [18,19,20], data comparing clinical outcomes in vaccinated and unvaccinated patients treated with mAb is unclear. Studies examining mAb treatment outcomes regarding vaccination status had conflicting results [21,22]. There remains a need to ascertain a difference in response to the mAb therapy between vaccinated and unvaccinated patients. These data may help prioritize patients if resources are scarce.

The goal of this study is to evaluate and compare the outcomes and risk of progression following mAb treatment between vaccinated and unvaccinated patients. We hypothesized that compared with unvaccinated patients, vaccinated patients would have a lower frequency of Emergency Department (ED) visits and hospitalizations within 14 days following mAb infusion, as well as a lower frequency of progression from mild to severe disease defined through the Intensive Care Unit (ICU) admissions within 14 days and deaths within 28 days from mAb treatment.

## 2. Materials and Methods

### 2.1. Setting and Cohort

This is a retrospective single-center study of 4128 COVID-19 positive patients treated with mAb under EUA between 1 March 2021 and 30 November 2021, under the guidance of the mAb Infusion Clinic at Spectrum Health.

Spectrum Health is a nonprofit quaternary-care health system based in Grand Rapids, Michigan, offering care to patients through 14 hospitals and 150 ambulatory clinics. In response to the mAb EUA in December 2020, Spectrum Health established a mAb Infusion Clinic. The role of the mAb Infusion Clinic was to coordinate mAb treatments systemwide. Any COVID-19 positive patient from our community could call the clinic to get evaluated for treatment; they did not need a referral, rather just proof of a positive test. Patients who tested for COVID-19 through our health system would receive positive test results through the patient portal and a message to call the clinic if they were interested in receiving treatment. All patients contacting the mAb Infusion Clinic would get evaluated for treatment. Eligible patients would receive mAb treatment in the mAb Infusion Clinic or a mobile unit, based on their physical location. The mobile unit was deployed across our region in response to changes in epidemiological data. Symptomatic patients who presented to the ED but were not admitted to the hospital received mAb in the ED under the mAb Infusion Clinic guidance. Per EUA requirements, all patient-related data were collected and stored in the mAb Infusion Clinic database. We compiled data from the mAb Infusion Clinic database and accessed patient Electronic Medical Records to ascertain the mAb treatment outcomes.

All patients 18 years and older who had a confirmed COVID-19 infection and were treated with mAb under EUA were eligible for this study. Included patients met EUA eligibility criteria defined as follows: confirmed COVID-19 infection, mild-to-moderate disease (no increase in oxygen requirement or need for a COVID-19 related hospitalization), early stage of the disease (within ten days of the symptom onset), weight at least 40 kg, and at risk for progression to severe disease, defined as a presence of at least one condition listed as a risk factor under EUA. Patients who received mAb for postexposure prophylaxis with no confirmed COVID-19 infection, those treated while hospitalized, or those under the age of 18 at the time of treatment were not eligible for this study. Additionally, patients with incomplete immunization histories were excluded.

**Figure 1:**
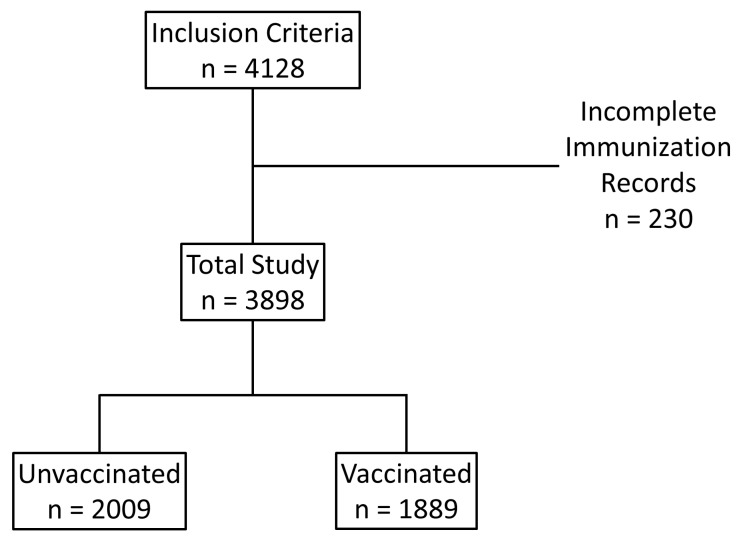
Patient Flow Diagram. Overall, 4128 patients met the inclusion criteria. From this, 230 patients were excluded due to incomplete immunization data. From 3898 patients included in the analysis, 1889 were vaccinated and 2009 were unvaccinated.

Of the 4128 patients meeting eligibility criteria, we excluded 230 patients due to incomplete immunization history (Figure 1). All 3898 patients included in the analysis were treated with a combination of Casirivimab and Imdevimab as a single intravenous infusion.

### 2.2. Vaccination Status

Patients who received at least two doses of the Pfizer or Moderna vaccines or at least one dose of the Johnson and Johnson vaccine were considered vaccinated. All other patients were defined as unvaccinated. Vaccine status was abstracted from the mAb Infusion Clinic Database.

### 2.3. Outcomes

We identified three clinically relevant outcomes: at least one ED visit within 14 days of mAb therapy, at least one hospital admission within 14 days of mAb infusion, and progression to severe disease, defined as an ICU admission within 14 days or death within 30 days of mAb administration. We abstracted data regarding ED visits, hospital admissions, ICU admissions, or reported death from patient Electronic Medical Records. We created binary variables for each outcome as the presence or absence of at least one ED visit, at least one hospital admission, and the presence or absence of progression to severe disease within the specified timeframes.

### 2.4. Covariates

We collected the following variables from the mAb Infusion Clinic database: patient age, gender, race, insurance or payer, comorbidities covered by the EUA, time from symptom onset to infusion, and comorbidities representing the risk factors for disease progression, as listed under EUA. We categorized the variables in the following way: patient sex (male, female), age (continuous), race (non-Hispanic white, Black or African American, Hispanic, Other, unknown/missing), insurance (Commercial, Medicaid, Medicare, Self-pay/Other/Unknown), infusion location (Infusion Clinic, Emergency Department/Observation, Mobile Unit), time from first symptoms to the infusion (typical from 0 to 7 Days, late from 7 to 10 Days), comorbidities (the type and number of comorbidities).

### 2.5. Statistical Analysis

We described the cohort using means (standard deviation) and frequencies (percentage). We calculated the absolute risk of the three clinical outcomes by dividing the number of patients with the outcome by the total number of patients eligible according to vaccination status. We used chi-square tests to assess the differences in the absolute risk of the three outcomes according to vaccination status. We used generalized linear regression models with a log link and a Poisson distribution with a robust variance estimator to estimate the relative risk of ED visits and hospital admission for the unvaccinated compared to the vaccinated patients treated with mAb according to the methods described by Zou [23]. We did not model the progression to severe disease outcome because the total number of events was very small. We specified one unadjusted model and one adjusted model for each of the two outcomes. The primary exposure was vaccination status, and we adjusted for time to infusion, insurance, infusion location, patient sex, patient age, patient race, and a count of the number of FDA eligibility comorbidities (1, 2, 3, and 4 or more). Beta-estimates from the models were exponentiated and reported as relative risk. We do not report covariate effect sizes; they are likely to be biased because models were not specified to address the relationships between covariates and the outcomes of interest [24]. Analyses were completed using SAS 9.4 (SAS Institute, Cary, NC, USA), and plots were generated using GGPlot2 [25] in R Studio version 022.2.2 (RStudio, PBC, Boston, MA, USA). We defined statistical significance as a *p*-value of less than or equal to 0.05.

## 3. Results

### 3.1. Cohorts Description

Of the 3898 patients included in the analysis, the majority were female (56.6%) and non-Hispanic Caucasians (90.4%). The mean age was 55.1 ± 16.4 years. Vaccination status was split nearly evenly, where 1889 (48.4%) patients were vaccinated, and 2009 (51.5%) were unvaccinated (Figure 1).

We noted several differences between vaccinated and unvaccinated patients. Vaccinated patients were older (mean age 58 ± 18.8 years vs. 52.4 ± 16.4 years), less racially diverse (92% vs. 89% of Caucasians), more often on Medicare (34.7% vs. 22.8%) and less frequently on Medicaid (4.1% vs. 11.1%) (Table 1).

The majority of both vaccinated and unvaccinated patients received mAb infusion within seven days from the symptom onset. However, unvaccinated patients were more likely to receive mAb after seven days from the symptom onset than vaccinated patients (23.3% vs. 18.8%, respectively). Unvaccinated patients were also 3.5 times more likely to receive mAb infusions in the ED (29.4% vs. 8.0%) (Table 1).

Vaccinated patients had a higher proportion of individual comorbidities with the exclusion of pregnancy. Pregnancy was more frequent in unvaccinated patients (1.6% vs. 0.7%, respectively). Vaccinated patients who received mAb infusion were generally in poorer health. Only 37.3% of vaccinated patients had zero to one comorbidity. In contrast, the majority of unvaccinated patients (62.7%) had only one comorbidity. Vaccinated patients also had a higher proportion of multiple comorbidities; in total, 64% of vaccinated patients had four or more comorbidities, compared with 36% of unvaccinated patients (Table 2).

### 3.2. Outcomes

We assessed the associations of vaccination status to ED visit within 14 days from mAb infusion hospital admission, within 14 days from mAb administration, and progression to severe disease, defined as an ICU admission within 14 days or all-cause mortality within 30 days from mAb treatment. Within the 14 days of mAb infusion, 296 (7.6%) patients visited ED at least once, and 154 (4.0%) patients were hospitalized. Twenty-five (0.6%) patients progressed to severe disease. Of those, 18 (0.46%) patients were admitted to the ICU. The overall mortality was twelve patients (0.3%).

All measured outcomes were significantly higher in the unvaccinated group with the exclusion of the 30-day mortality. Unvaccinated patients had significantly more hospitalizations (5.8% vs. 2%, *p* < 0.0001) and ED visits than vaccinated patients (10.8% vs. 4.2%, *p* < 0.0001) within 14 days of mAb infusion. Progression to severe disease was also significantly higher in unvaccinated patients (0.9% vs. 0.3%, *p* = 0.016). Unvaccinated patients had significantly more ICU admissions (0.8% vs. 0.1%, *p* = 0.015). There were eight deaths (0.4%) in the unvaccinated group and four (0.2%) among vaccinated patients (*p* = 0.2543) (Table 3).

**Figure 2:**
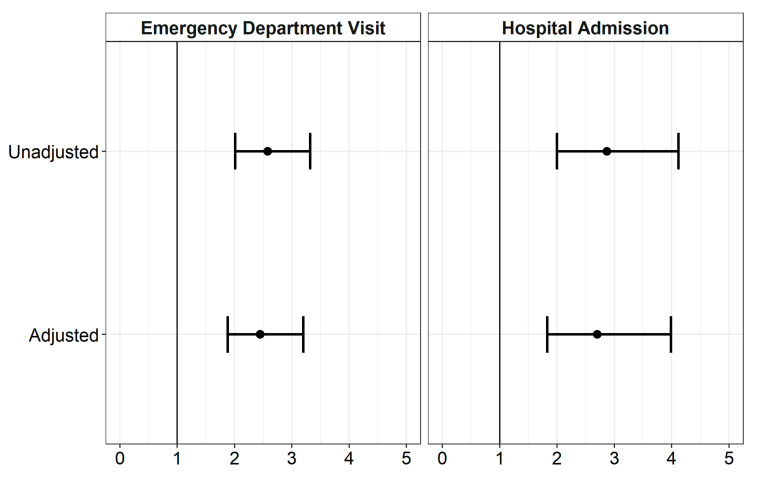
Unadjusted and adjusted relative risk estimates with 95% confidence intervals for emergency department (ED) and hospital admission among vaccinated and unvaccinated patients treated with monoclonal antibody therapy. The primary exposure was vaccination status. The adjusted models included the following variables: time to infusion, insurance, infusion location, patient sex, age, race, and a count of comorbidities (1, 2, 3, and 4 or more). Relative risk estimates were calculated by exponentiating beta estimates from modified Poisson models with a robust standard error estimator.

Unadjusted and adjusted modified Poisson regression models found that vaccination status was independently associated with both ED visits and hospital admissions within 14 days of mAb infusion. Unvaccinated patients had an almost three-fold higher risk of ED visits (RR, 2.87; 95% CI, 2.00–4.12) and a 2.5-fold higher risk of hospital admissions (RR, 2.58; 95% CI, 2.01–3.32) within 14 days from mAb infusion compared to vaccinated patients in unadjusted models. When we adjusted the models for insurance, infusion location, patient sex, patient age, patient race, time to infusion, and a count of the number of FDA eligibility comorbidities (0, 1, 2, 3, and 4 or more), we found the risk of ED visits for unvaccinated patients was 2.5-fold (RR, 2.45; 95% CI, 1.88–3.20) higher compared to vaccinated, and the risk of hospital admissions for unvaccinated patients was 2.7-fold (RR, 2.70; 95% CI, 1.88–3.99) greater (Figure 2; Appendix A Table A1).

## 4. Discussion

Our findings suggest that following mAb therapy, compared with vaccinated, unvaccinated COVID-19 patients have a higher risk of progression to severe disease, demonstrated by the significantly higher risks of ED visits and hospitalizations.

The vaccinated group was older, had a higher prevalence of all individual comorbidities except pregnancy, and had a higher number of comorbidities per patient, thus placing them at a higher risk for progression to severe disease. Despite this, fewer vaccinated patients utilized hospital resources and progressed to severe disease. Vaccine status was an independent predictor of ED and hospital utilization. These data suggest that primary prevention through vaccination is essential for reducing the risk of adverse clinical outcomes, even with mAb treatment.

Several studies have addressed the potential relationship between vaccination status and mAb efficacy but with discordant results. In a single-center study of 1222 patients, Zitek et al. found that vaccination status significantly decreased the odds of hospitalization within 28 days of mAb infusion but not the odds of ED presentation. This study had an uneven distribution of vaccinated and unvaccinated patients (16.2% vs. 79.3%, respectively), and did not evaluate risk factors distribution in the groups [20]. Bierle et al. noted decreased frequency of hospitalization in both vaccinated and unvaccinated patients following mAb treatment compared with untreated controls. However, they did not demonstrate a significant difference in response to mAb treatment between vaccinated and unvaccinated patients, likely because of a small sample—from 112 treated patients, 55 were vaccinated [11]. In another smaller study of 250 patients, Guo et al. failed to find a statistically significant difference in the frequency of hospitalization following mAb infusion between vaccinated and unvaccinated patients (5% vs. 7%, respectively). This study included a younger patient population with fewer comorbidities and had an uneven distribution between vaccinated (*n* = 162) and unvaccinated (*n* = 8) patients [21]. Keshishian et al. performed a single-center study that included 743 patients, with the majority (585) being unvaccinated. Seventeen patients were admitted to the hospital, with no significant difference between the vaccinated and the unvaccinated. In contrast to our study, vaccinated patients were more likely to present to the ED and urgent care within 28 days. However, Keshishian et al. examined urgent care presentation and had a longer follow-up period [22]. Srinivasan et al. performed a multi-site retrospective review of 2209 patients treated outpatient with mAb in California over one year. Their results demonstrated a protective effect of mAb in unvaccinated but not in vaccinated high-risk patients when evaluating a compositive endpoint of 30-day incidence of ED visit, hospitalization, or death. The use of a composite outcome may have led to the observed difference in the outcomes between this and our study [26]. A large retrospective study by Douin et al. examined charts of 7406 patients. It demonstrated an odds ratio of 2.76 for the development of treatment failure, defined as hospitalization or death within 28 days of mAb therapy, for unvaccinated patients compared to vaccinated patients, which aligns with our results [27].

Most patients treated in our clinic belong to the Greater Grand Rapids area, including Kent, Ottawa, Montcalm, and Ionia counties, with 22.22% of the population being non-Caucasians [28]. Even though the COVID-19 pandemic disproportionately impacted communities of color in Michigan [29,30], only 9.8% of patients treated in our clinic were non-Caucasians, suggesting possible racial and ethnic disparities. Race is a known risk factor for severe COVID-19 disease [31,32]. The unvaccinated group contained a higher proportion of people of color (8.7% vs. 5.9%). In addition, unvaccinated patients were more likely to receive mAb therapy in the ED than in the infusion clinic. This suggests that the unvaccinated patients may have been sicker at the time of infusion or lacked access to healthcare resources needed to obtain mAb therapy in the infusion clinic. These scenarios may also not be mutually exclusive. At the very least, the demographics suggest a need for health equity promotion to reduce observed health inequalities.

Our study has several limitations. It represents a single-site experience. All patients were treated with a single mAb agent. Due to the timeline, we anticipate delta as the primary variant. Whether our data can be generalized to the other mAb and SARS-CoV-2 variants is yet to be determined. Additionally, we included all-cause ED visits, hospital admissions, and mortality in the analysis, understanding that the reason for patient presentation may not be related to COVID-19 disease progression. However, considering the baseline health of the groups, we speculate that vaccinated patients would have a higher frequency of non-COVID-19 related visits. This further supports our finding that mAb provides better protection to vaccinated patients. Furthermore, our comorbidity data were limited to conditions listed under EUA as risk factors for COVID-19 disease progression. We may have inadvertently excluded specific conditions which predisposed patients to the progression of COVID-19. Observational studies are always at risk for unmeasured and residual confounding, and the baseline covariates included in this study were not evenly distributed between the vaccinated and unvaccinated patients. There are multiple methods for addressing baseline imbalance in known confounders, including propensity score methods (PSM). However, it has been shown that covariate adjustment is an acceptable method for adjustment, and often outperforms PSM with regard to precision and bias [33,34]. Lastly, our counts for severe progression were too small to be assessed in models and may limit the ability to accurately determine the likelihood of progression to severe disease.

These limitations are counterbalanced by a large cohort with an even distribution of vaccinated and unvaccinated patients and a detailed analysis that includes patients’ demographics, insurance, and comorbidities.

## 5. Conclusions

Vaccinated COVID-19-positive patients treated with mAb had a significantly lower risk of subsequent ED visits and hospitalizations than unvaccinated patients treated with mAb. Continued emphasis on global immunization, mAb administration to at-risk patients, and equitable access to both are critical to reducing the burden of COVID-19 on hospital systems. In addition, further prospective controlled studies are needed to confirm the added benefit between COVID-19 vaccines and mAb infusion as COVID-19 evolves and new variants of concern emerge.

## Figures and Tables

**Table 1:** Patient Characteristics.

	Total *n* = 3898	Unvaccinated *n* = 2009	Vaccinated *n* = 1889
Sex, *n* (%)			
Male	1692 (43.1)	862 (42.9)	830 (43.9)
Female	2206 (56.4)	1147 (57.0)	1059 (56.1)
Age, years ^	55.1 ± 16.4	52.4 ± 16.4	58.0 ± 18.8
Race, *n* (%)			
Non-Hispanic Caucasian	3525 (90.4)	1783 (88.7)	1742 (92.2)
Black or African American	111 (2.9)	72 (3.6)	39 (2.1)
Hispanic	99 (2.5)	62 (3.1)	37 (2.0)
Other	74 (1.9)	40 (2)	34 (1.8)
Unknown/Missing	89 (2.3)	52 (2.6)	37 (2.0)
Insurance, *n* (%)			
Commercial	2209 (56.7)	1147 (57.1)	1062 (56.2)
Medicaid	301 (7.7)	223 (11.1)	78 (4.1)
Medicare	1115 (28.6)	459 (22.8)	656 (34.7)
Self-pay/Other/Unknown	273 (7.0)	180 (9.0)	93 (4.9)
Infusion Location, *n* (%)			
Infusion Clinic	2876 (73.4)	1264 (62.9)	1612 (85.3)
Emergency Department/Observation	741 (19.0)	590 (29.4)	151 (8.0)
Mobile Unit/In-home	281 (7.2)	155 (7.7)	126 (6.7)
Infusion Prior to First Symptoms *, *n* (%)			
≤7 Days	3074 (78.9)	1540 (76.7)	1534 (81.2)
>7 Days	824 (21.1)	469 (23.3)	355 (18.8)

^ Mean ± Standard Deviation. * Patient Reported.

**Table 2:** Comorbidities, *n* (%).

	Unvaccinated *n* = 2009	Vaccinated *n* = 1889
Elevated BMI * (>35 kg/m^2^)	1441 (71.7)	1446 (76.5)
Hypertension	527 (26.2)	775 (41.0)
Smoker	547 (27.2)	550 (29.1)
Lung Disease	336 (16.7)	402 (21.3
Cardiovascular Disease	243 (12.1)	380 (20.1)
Diabetes	222 (11.1)	324 (17.2)
Cancer	118 (5.9)	200 (13.8)
Immunosuppression	114 (7.2)	147 (7.8)
Chronic Kidney Disease	70 (3.5)	119 (6.3)
Neurological Condition	67 (3.3)	79 (4.2)
Pregnancy	33 (1.6)	13 (0.7)
Transplant	9 (0.4)	27 (1.4)
Other	34 (1.7)	45 (2.4)
Number of Comorbidities per Patient ^		
One	940 (46.8)	560 (29.6)
Two	569 (28.3)	566 (30.0)
Three	298 (14.8)	404 (21.4)
Four or more	202 (10.1)	359 (19.0)

^ Patients may have multiple comorbidities. * BMI (Body Mass Index).

**Table 3:** Patient Outcomes, *n* (%).

	Total *n* = 3898	Unvaccinated *n* = 2009	Vaccinated *n* = 1889	*p*-Value
Hospital Admissions	154 (4.0)	116 (5.8)	38 (2.0)	<0.0001
Emergency Department Visit	296 (7.6)	217 (10.8)	79 (4.2)	<0.0001
Progression to Severe Disease	25 (0.6)	19 (0.9)	6 (0.3)	0.016
ICU Admission	18 (0.46)	16 (0.8)	2 (0.1)	0.015
30-Day Mortality	12 (0.3)	8 (0.4)	4 (0.2)	0.2543

Hospital admissions within 14 days of infusion. Emergency Department visit within 14 days of infusion. Progression to severe disease is defined as ICU (Intensive Care Unit) admission within 14 days or death within 30 days of infusion. ICU admission within 14 days of Infusion. 30-Day Mortality following infusion.

## Data Availability

Aggregated data are available upon written request to the corresponding author. Non-aggregated data are not publicly available as they may contain information that may compromise the privacy of research participants.

## Appendix A

**Table A1:** Relative risk estimates from modified Poisson regression models that correspond to Figure 2.

Outcome	Unadjusted (95% CI)	Adjusted (95% CI)
Emergency Department Visit	2.58 (2.01, 3.32)	2.45 (1.88, 3.20)
Hospital Admissions	2.87 (2.00, 4.12)	2.70 (1.83, 3.99)
CI: Confidence Interval		

Unadjusted and adjusted relative risk estimates with 95% confidence intervals for emergency department visits and hospital admission among vaccinated and unvaccinated patients treated with monoclonal antibody therapy. The primary exposure was vaccination status. The adjusted models included the following variables: time to infusion, insurance, infusion location, patient sex, age, race, and a count of comorbidities (1, 2, 3, and 4 or more). Relative risk estimates were calculated by exponentiating beta estimates from modified Poisson models with a robust standard error estimator.

## References

[1] Cucinotta D., Vanelli M. (2020). WHO Declares COVID-19 a Pandemic. Acta Biomed..

[2] Determination that a Public Health Emergency Exists (hhs.gov). https://aspr.hhs.gov/legal/PHE/Pages/2019-nCoV.aspx.

[3] U.S. Department of Health and Human Services Notice of Emergency Use Authorization Declaration. (fda.gov). Emergency Use Authorization|FDA. https://www.fda.gov/emergency-preparedness-and-response/mcm-legal-regulatory-and-policy-framework/emergency-use-authorization.

[4] U.S. Food and Drug administration Coronavirus (COVID-19) Update: FDA Issues Emergency Use Authorization for Potential COVID-19 Treatment (fda.gov). https://www.fda.gov/news-events/press-announcements/coronavirus-covid-19-update-fda-issues-emergency-use-authorization-potential-covid-19-treatment.

[5] U.S. Food and Drug Administration Coronavirus (COVID-19) Update: FDA Authorizes Monoclonal Antibody for Treatment of COVID-19.(fda.gov). www.fda.gov/news-events/press-announcements/coronavirus-covid-19-update-fda-authorizes-monoclonal-antibody-treatment-covid-19.

[6] U.S. Food and Drug Administration Fact Sheet for Healthcare Providers. Emergency Use Authorization (EUA) for Casirivmab and Imdevimab. Regeneron EUA HCP Fact Sheet 01242022 (fda.gov). https://www.fda.gov/media/145611/download.

[7] U.S. Food and Drug Administration Fact Sheet for Healthcare Providers. Emergency Use Authorization (EUA) of Bamlanivimab and Etesevimab. https://www.fda.gov/media/145802/download.

[8] U.S. Food and Drug Administration Fact Sheet for Healthcare Providers. Emergency Use Authorization (EUA) of Sotrovimab. https://www.fda.gov/media/149534/download.

[9] Weinreich D.M., Sivapalasingam S., Norton T., Ali S., Gao H., Bhore R., Xiao J., Hooper A.T., Hamilton J.D., Musser B.J. (2021). REGEN-COV antibody combination and outcomes in outpatients with COVID-19. N. Engl. J. Med..

[10] Dougan M., Nirula A., Azizad M., Mocherla B., Gottlieb R.L., Chen P., Hebert C., Perry R., Boscia J., Heller B. (2021). Bamlanivimab plus etesevimab in mild or moderate COVID-19. N. Engl. J. Med..

[11] Bierle D.M., Ganesh R., Razonable R.R. (2021). Breakthrough COVID-19 and casirivimab-imdevimab treatment during a SARS-CoV-2 B1. 617.2 (Delta) surge. J. Clin. Virol..

[12] Food and Drug Administration (2020). Pfizer-BioNTech COVID-19 Vaccine Emergency Use Authorization.

[13] Food and Drug Administration (2020). Moderna COVID-19 Vaccine Emergency Use Authorization.

[14] Food and Drug Administration (2021). Janssen COVID-19 Vaccine Emergency Use Authorization.

[15] Haas E.J., Angulo F.J., McLaughlin J.M., Anis E., Singer S.R., Khan F., Brooks N., Smaja M., Mircus G., Pan K. (2021). Impact and effectiveness of mRNA BNT162b2 vaccine against SARS-CoV-2 infections and COVID-19 cases, hospitalisations, and deaths following a nationwide vaccination campaign in Israel: An observational study using national surveillance data. Lancet.

[16] Tenforde M.W., Patel M.M., Ginde A.A., Douin D.J., Talbot H.K., Casey J.D., Mohr N.M., Zepeski A., Gaglani M., McNeal T. (2022). Effectiveness of severe acute respiratory syndrome coronavirus 2 messenger RNA vaccines for preventing coronavirus disease 2019 hospitalizations in the United States. Clin. Infect. Dis..

[17] Tenforde M.W., Self W.H., Naioti E.A., Ginde A.A., Douin D.J., Olson S.M., Talbot H.K., Casey J.D., Mohr N.M., Zepeski A. (2021). Sustained effectiveness of Pfizer-BioNTech and Moderna vaccines against COVID-19 associated hospitalizations among adults—United States, March–July 2021. Morb. Mortal. Wkly. Rep..

[18] Hirotsu Y., Tsutsui T., Kakizaki Y., Miyashita Y., Iwase F., Maejima M., Sugiura H., Mochizuki H., Omata M. (2021). Active immunization by COVID-19 mRNA vaccine results in rapid antibody response and virus reduction in breakthrough infection by Delta (B. 1.617. 2). Res. Sq..

[19] Bierle D.M., Ganesh R., Tulledge-Scheitel S., Hanson S.N., Arndt L.L., Wilker C.G., Razonable R.R. (2022). Monoclonal antibody treatment of breakthrough COVID-19 in fully vaccinated individuals with high-risk comorbidities. J. Infect. Dis..

[20] Zitek T., Jodoin K., Kheradia T., Napolillo R., Dalley M.T., Quenzer F., Farcy D.A. (2022). Vaccinated patients have reduced rates of hospitalization after receiving casirivimab and imdevimab for COVID-19. Am. J. Emerg. Med..

[21] Guo Y., Cowman K., Chang M., Bao H., Golia A., Mcsweeney T., Bard L., Simpson R., Andrews E., Pirofski L.-A. (2022). Assessment of unvaccinated and vaccinated patients with coronavirus disease 2019 (COVID-19) treated with monoclonal antibodies during the delta wave (July 1–August 20, 2021): A retrospective observational monocentric study. BMC Infect. Dis..

[22] Keshishian E., Kuge E., Memmott J., Hasenbalg P., Belford N., Matlock A., Schritter S., Agbayani G., Dietrich T., Santarelli A. (2022). Casirivimab/imdevimab treatment for outpatient COVID-19 during a SARS-CoV-2 B1.617.2 (Delta) surge at a community hospital. J. Osteopath. Med..

[23] Zou G. (2004). A Modified Poisson Regression Approach to Prospective Studies with Binary Data. Am. J. Epidemiol..

[24] Westreich D., Greenland S. (2013). The Table 2 fallacy: Presenting and interpreting confounder and modifier coefficients. Am. J. Epidemiol..

[25] Wickham H. (2016). ggplot2: Elegant Graphics for Data Analysis.

[26] Srinivasan V., Weinstein S.E., Bhimani A., Clemons N.C., Dinolfo M., Shin C.S., Grier J., Lopez A., Braggs J., Boucher J. (2022). On Variants and Vaccines: The Effectiveness of COVID-19 Monoclonal Antibody Therapy during Two Distinct Periods in the Pandemic. PLoS ONE.

[27] Douin D.J., Wogu A.F., Beaty L.E., Carlson N.E., Bennett T.D., Aggarwal N.R., Mayer D.A., Ong T.C., Russell S., Steele J. (2022). Association between Treatment Failure and Hospitalization after Receipt of Neutralizing Monoclonal Antibody Treatment for COVID-19 Outpatients. BMC Infect. Dis..

[28] Population and Demographics|Greater Grand Rapids The Right Place. www.rightplace.org/why-greater-grand-rapids/talent/demographics.

[29] Anderson-Carpenter K.D., Neal Z.P. (2022). Racial disparities in COVID-19 impacts in Michigan, USA. J. Racial Ethn. Health Disparities.

[30] Raifman M.A., Raifman J.R. (2020). Disparities in the population at risk of severe illness from COVID-19 by race/ethnicity and income. Am. J. Prev. Med..

[31] Hooper M.W., Nápoles A.M., Pérez-Stable E.J. (2020). COVID-19 and racial/ethnic disparities. JAMA.

[32] Selden T.M., Berdahl T.A. (2020). COVID-19 And Racial/Ethnic Disparities in Health Risk, Employment, and Household Composition: Study examines potential explanations for racial-ethnic disparities in COVID-19 hospitalizations and mortality. Health Aff..

[33] Elze M.C., Gregson J., Baber U., Williamson E., Sartori S., Mehran R., Nichols M., Stone G.W., Pocock S.J. (2017). Comparison of Propensity Score Methods and Covariate Adjustment: Evaluation in 4 Cardiovascular Studies. J. Am. Coll. Cardiol..

[34] Cepeda M.S., Boston R., Farrar J.T., Strom B.L. (2003). Comparison of logistic regression versus propensity score when the number of events is low and there are multiple confounders. Am. J. Epidemiol..

